# Albumin infusion rate and plasma volume expansion: a randomized clinical trial in postoperative patients after major surgery

**DOI:** 10.1186/s13054-019-2477-7

**Published:** 2019-05-28

**Authors:** Svajunas Statkevicius, Johan Bonnevier, Jane Fisher, Björn P. Bark, Erik Larsson, Carl M. Öberg, Päivi Kannisto, Bobby Tingstedt, Peter Bentzer

**Affiliations:** 1 Department of Anesthesia & Intensive Care, Skåne University Hospital, Lund, Sweden; 2 Department of Infectious Diseases, Skåne University Hospital, Lund, Sweden; 3 Department of Radiation Physics, Skåne University Hospital, Lund, Sweden; 4 Department of Nephrology, Skåne University Hospital, Lund, Sweden; 5 Department of Gynecology and Obstetrics, Skåne University Hospital, Lund, Sweden; 6 Department of Surgery, Skåne University Hospital, Lund, Sweden; 7 Department of Anesthesia and Intensive Care, Helsingborg Hospital, Helsingborg and Lund University, 251 87 Helsingborg, Sweden; 8 Department of Clinical Sciences, Anesthesiology, Lund University, Lund, Sweden

**Keywords:** Plasma volume expanders, Fluid therapy, Serum albumin

## Abstract

**Background:**

Optimal infusion rate of colloids in patients with suspected hypovolemia is unknown, and the primary objective of the present study was to test if plasma volume expansion by 5% albumin is greater if fluid is administered slowly rather than rapidly.

**Methods:**

Patients with signs of hypoperfusion after major abdominal surgery were randomized to intravenous infusion of 5% albumin at a dose of 10 ml/kg (ideal body weight) either rapidly (30 min) or slowly (180 min). Plasma volume was measured using radiolabeled albumin at baseline, at 30 min, and at 180 min after the start of infusion. Primary outcome was change in plasma volume from the start of infusion to 180 min after the start of infusion. Secondary outcomes included the change in the area under the plasma volume curve and transcapillary escape rate (TER) for albumin from 180 to 240 min after the start of albumin infusion.

**Results:**

A total of 33 and 31 patients were included in the analysis in the slow and rapid groups, respectively. The change in plasma volume from the start of infusion to 180 min did not differ between the slow and rapid infusion groups (7.4 ± 2.6 vs. 6.5 ± 4.1 ml/kg; absolute difference, 0.9 ml/kg [95%CI, − 0.8 to 2.6], *P* = 0.301). Change in the area under the plasma volume curve was smaller in the slow than in the rapid infusion group and was 866 ± 341 and 1226 ± 419 min ml/kg, respectively, *P* < 0.001. TER for albumin did not differ and was 5.3 ± 3.1%/h and 5.4 ± 3%/h in the slow and in the rapid infusion groups, respectively, *P* = 0.931.

**Conclusions:**

This study does not support our hypothesis that a slow infusion of colloid results in a greater plasma volume expansion than a rapid infusion. Instead, our result of a smaller change in the area under the plasma volume curve indicates that a slow infusion results in a less efficient plasma volume expansion, but further studies are required to confirm this finding. A rapid infusion has no effect on vascular leak as measured after completion of the infusion.

**Trial registration:**

EudraCT2013-004446-42 registered December 23, 2014.

**Electronic supplementary material:**

The online version of this article (10.1186/s13054-019-2477-7) contains supplementary material, which is available to authorized users.

## Background

Major surgery initiates a systemic inflammatory response syndrome which disrupts the normal regulation of transcapillary fluid exchange and may induce tissue edema and hypovolemia [1]. Fluid replacement is therefore a cornerstone in the perioperative treatment of these patients as well as in patients suffering from increased vascular leakage of other etiologies. Although fluid therapy is life-saving, it is also associated with side effects such as further edema formation and compartment syndromes which may adversely affect the outcome [2–8].

From a clinical perspective, it is therefore important that fluid administered to counteract hypovolemia is retained intravascularly as much as possible. Colloids are macromolecules for which the vessel wall has a low permeability, and less volume is required for an equal plasma volume expansion compared to crystalloids [9–11]. However, extravasation of colloids is not only a function of the vessel wall permeability but is also dependent on the volume of fluid that is filtered across the capillary wall, which in turn depends on the transcapillary hydrostatic pressure [12]. This indicates that infusion rate may influence extravasation of colloids by effects on hydrostatic pressure, but the importance of such an effect on plasma volume expansion by a colloid is unclear. The hypothesis is supported by studies in rodent models of sepsis showing that plasma volume expansion in experimental sepsis is greater after a slow infusion compared to a rapid infusion of the same volume of colloid [13, 14]. In contrast, no effect of a high infusion rate on extravasation of albumin could be demonstrated in porcine endotoxemia [15].

Recent surveys show that infusion rate of resuscitation fluids is highly variable [16], and no study has addressed the importance of infusion rates for plasma volume expansion in a clinical setting. While rapid correction of suspected hypovolemia makes intuitive sense, the recent FEAST trial in septic children showed a surprising increase in mortality following resuscitation using fluid boluses compared to less aggressive fluid resuscitation [17]. Moreover, rapid plasma volume expansion has been suggested to induce shedding of the endothelial glycocalyx through the release of atrial natriuretic peptide [18]. Taken together, the above suggest that rapid infusion of resuscitation fluids may have adverse effects and highlight the need for further knowledge in this aspect of fluid resuscitation.

Based on these considerations, the primary objective of this study was to test the hypothesis that plasma volume expansion by a given volume of colloid is greater if fluid is administered slowly rather than rapidly. For this purpose, postoperative patients with suspected hypovolemia were randomized to receive 5% albumin at a dose of 10 ml/kg in 30 min or in 180 min. Primary outcome was change in plasma volume from baseline to 180 min after the start of the albumin infusion. Secondary endpoints included the change in the area under the plasma volume curve from the start of infusion to 180 min after the start of the infusion, transcapillary escape rate of albumin, and postoperative morbidity. To assess the effects of the intervention on the endothelial glycocalyx, we also measured the plasma concentration of glycocalyx components and hormones involved in fluid balance.

## Methods

We conducted a single-center, prospective randomized physiological trial of albumin administration at two different infusion rates to patients with suspected hypovolemia following major abdominal surgery. The study was approved by the regional ethical vetting board (# 2014/15) and was conducted at Skåne University Hospital, Lund, Sweden. Written informed consent was obtained from all subjects. The protocol was amended two times. The first amendment extended the inclusion criteria to include postoperative patients after major gynecological cancer surgery (open ovarian and endometrial debulking surgery) to promote recruitment. The second amendment changed the interim analysis plan by adding the Haybittle-Peto boundary for the testing of efficacy. In addition, vasopressor and inotropic therapy were omitted as exclusion criteria. The first amendment was performed after 1 patient had been included, and the second was performed after 24 patients had been included. The trial was registered in the European Clinical Trials Database (EudraCT 2013-004446-42) and on ClinicalTrials.gov (NCT02728921). The trial was authorized by the Swedish Medical Products Agency to proceed on 9 February 2014. The experimental protocol has been published [19].

### Inclusion criteria

Patients scheduled for non-emergent Whipple operation or major gynecological cancer surgery ≥ 40 years of age were approached by a member of the research team. The patients were informed about the possibility to participate in the study after admission to the postoperative care unit, should they fulfill the postoperative inclusion criteria. Written informed consent was collected from interested patients. Patients who had given a written consent prior to the operation were screened by a member of the research team the first 5 h after admission to the post-anesthesia care unit. The inclusion criteria were (1) written consent and (2) indication for fluid therapy as judged by the physician caring for the patient and at least one of the following criteria: (a) positive “leg raising test” (pulse pressure increase > 9%) [20, 21], (b) central venous oxygen saturation < 70%, (c) arterial lactate > 2.0 mmol/l, (d) urine output < 0.5 ml/kg the hour prior to inclusion, (e) respiratory variation of the inferior vena cava of more than 15% as measured by ultrasound [22, 23], and (f) systolic blood pressure < 100 mmHg or mean arterial blood pressure < 55 mmHg.

### Exclusion criteria

Patients fulfilling any of the following criteria were excluded: (1) hypersensitivity to the active drug or the tracer; (2) signs of postoperative bleeding; (3) history of heart failure; (4) the physician caring for the patient considered that there were strong reasons to administer another fluid or the same fluid, but in another way or in a different volume than stated in the protocol; (5) pregnancy; and (6) clinical judgment by the investigator or the treating physician that the patient should not participate in the study for reasons other than described above. Predefined permanent withdrawal criteria included the change of surgical procedure or the occurrence of serious adverse events.

#### Intra- and postoperative care of the patients

Included patients received routine pre- and intraoperative care. Anesthesia was induced intravenously using propofol and maintained using either sevoflurane or desflurane. Patients received an epidural catheter for intra- and postoperative analgesia unless contraindicated. Postoperative epidural analgesia was provided using bupivacaine (2.5 mg/ml) and morphine (0.05 mg/ml) at a rate of 4–6 ml/h. Crystalloids and colloids were used as resuscitation fluids intraoperatively at the discretion of the attending anesthetist. A hemoglobin level of 80–90 g/l was the transfusion trigger. Analgesics were given as needed during the intervention phase of the study, and the rate of vasoactive agents was adjusted to maintain mean arterial pressure > 65 mmHg. No fluids other than maintenance (2.5% or 5% glucose with electrolytes at a rate of 1 ml/kg/h) and study fluids were given during the intervention period. The trial was audited by external monitors and auditing included source data verification.

### Intervention

Eligible patients were randomized at a ratio of 1:1 using sealed envelopes to receive 5% albumin at a dose of 10 ml/kg of predicted body weight [24] in either 30 min or 180 min. Sealed envelopes were prepared by an independent party (Clinical Research Unit, Skåne University Hospital, Lund). Randomization was performed using a computerized random number generator, and the research team was blinded to block size.

### Outcomes

The primary outcome was change in plasma volume from the start to 180 min after the start of albumin infusion. Secondary outcomes were change in the area under the plasma volume curve from the start to 180 min after the start of infusion of albumin, transcapillary escape rate (TER) for albumin, changes in hemodynamic parameters, diuresis, plasma concentration of hormones involved in fluid homeostasis, and plasma concentration of glycocalyx components and incidence of postoperative complications up to 30 days postoperatively (see Additional file 1: Table S1 for the definitions of complications).

### Measurements

Plasma volume was measured by calculating the distribution volume of ^125^I-human serum albumin (SERALB-125®, CIS Bio International, Gif-Sur-Yvette, France) administered intravenously before the start of the albumin infusion, at 30 min, and at 180 min after the start of the infusion. The method is considered to be the gold standard for the measurement of plasma volume [25] and is described in more detail in the supplement. The change in the area under the plasma volume curve was calculated using the trapezoid rule. Transcapillary escape rate for albumin is a measure of leakage of albumin from microvessels into the interstitium and was measured by measuring plasma concentration of ^125^I-human serum albumin at 10, 30, 45, and 60 min after the last injection of the tracer (Additional file 1: Figure S1). The decrease in plasma concentration of ^125^I-human serum albumin was fitted to be a mono exponential function and is expressed as the percentage of the decrease in plasma concentration of ^125^I-human serum albumin per hour [26–28]. Potential sources of error in measurement of transcapillary escape rate for albumin and plasma volume have been evaluated previously and have been found to be small [19, 26, 29]. Transcapillary escape rate was measured between 180 and 240 min after the start of the infusion to ensure that both groups had received the same dose of albumin when the measurement was performed.

Plasma concentrations of glypican-4 (Cloud-Clone Corp), hyaluronan (Echelon Biosciences), Syndecan-1 (Diaclone), renin (IDS), copeptin (Brahms GmbH), albumin (Roche), and Mid Regional-pro Atrial Natriuretic Peptide (MR-proANP) (Brahms GmbH) were determined by immunologic assays according to the manufacturer’s instructions before and at 180 min after the start of the albumin infusion. On a post hoc basis, we analyzed albumin before and at 180 min after the start of the albumin infusion using an immunological assay (Roche). Blood gas analysis and determination of hematocrit and plasma lactate were performed using a blood gas analyzer (Radiometer 850, Radiometer). Hematocrit was measured every 30 min from baseline to 180 min after the start of infusion. The participants received routine postoperative care after the study protocol was completed. Measurement of plasma volumes, transcapillary escape rate, and plasma concentrations of glycocalyx components and hormones were made blinded to treatment. Baseline hemodynamic data and blood gases were collected prior to treatment allocation whereas these parameters were recorded by an investigator aware of the treatment allocation at the later time points. For an overview of the measurements, see Additional file 1: Figure S1.

### Data collection and management

Comorbidities, medications, routine laboratory analysis results, American Society of Anesthesiology (ASA) classification, and Revised Cardiac Risk Index were collected from the hospital electronic chart system or calculated. Perioperative data were collected from the anesthesia chart. Hemodynamic data were recorded immediately prior to the start and at 180 min after the start of albumin infusion. Diuresis was recorded 4 h before and 6 h after the start of infusion.

### Statistical analysis

In experimental studies, a 6-ml/kg greater plasma volume expansion was found after slow administration of 5% albumin at a dose of 12 ml/kg compared with a bolus dose of the same volume [13, 14]. Based on these data, the present study was powered to detect a difference in plasma volume expansion between the groups of 4 ml/kg. Assuming a standard deviation of the change in plasma volume of 5 ml/kg [30], about 30 patients in each group were required to obtain a power of 80% using an unpaired *t* test. To adjust for the possibility that patients did not complete the protocol, we aimed to include 35 patients in each arm.

An interim analysis for the assessment of efficacy was performed after 36 patients had completed the protocol. The Haybittle-Peto boundary was used to test for efficacy meaning that if a difference with regard to the primary endpoint with a *P* ≤ 0.001 was detected, the study could be stopped.

Statistical analyses were performed per protocol by an independent statistician blinded to the treatment allocation, and the main results were interpreted as specified in the protocol [19]. Hypothesis testing was made using two-tailed testing, and a *P* value below 0.05 was considered significant. Assessment of normality was performed using histograms and the Shapiro-Wilk test and followed by the unpaired *t* test or the Mann-Whitney test as appropriate. Fisher’s exact test was used for categorical variables. Analysis of covariance (ANCOVA) and Pearson correlation analysis were used for the sensitivity analysis. Influence of baseline blood volume and type of surgery on treatment effect were assessed in pre-specified sensitivity analyses. Data are presented as median and interquartile range or as mean ± SD. SAS 9.4 Institute Inc., Cary, NC, USA, was used for the analysis.

## Results

### Patients

**Fig. 1:**
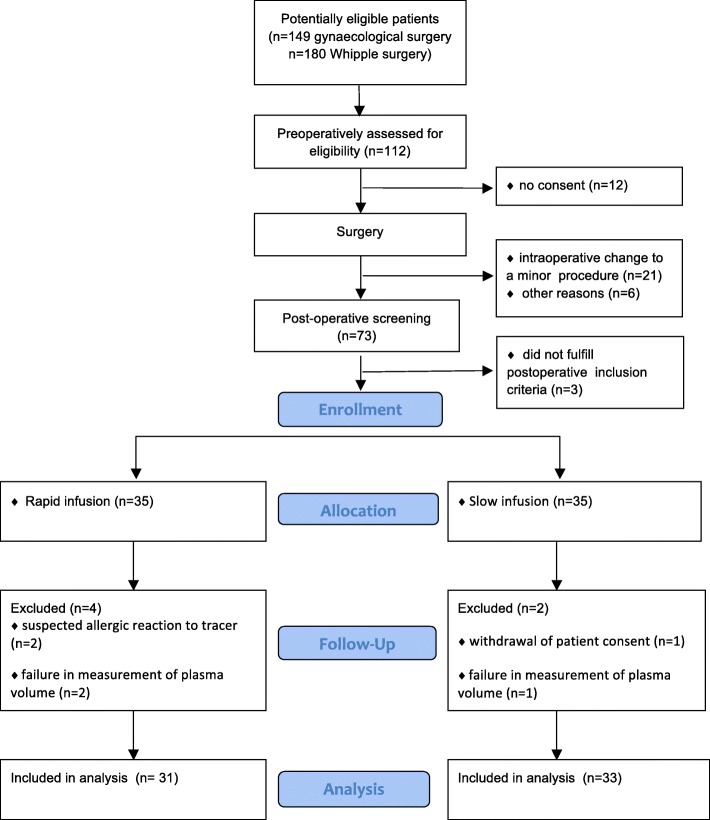
CONSORT flowchart

**Fig. 2:**
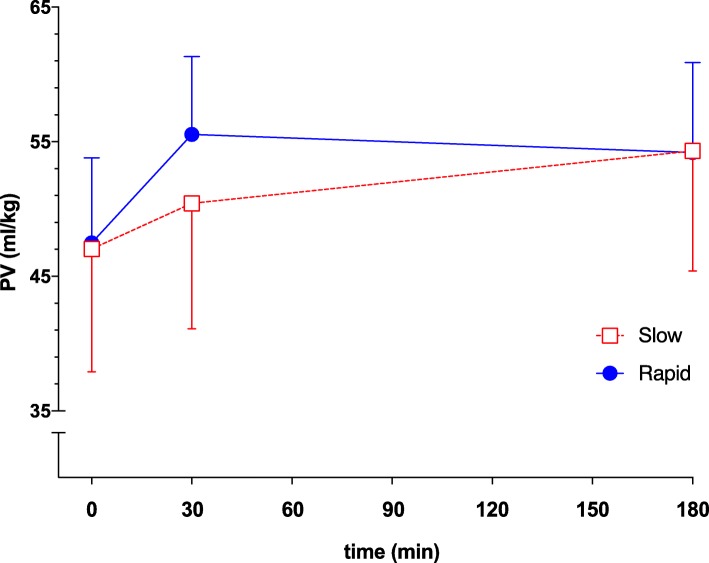
Plasma volumes (PV, ml/kg) in the slow and rapid infusion groups. Data are shown as mean and SD. Error bars for the rapid group points up, and error bars for the slow group points down

A total of 70 patients were enrolled between the 18 June 2014 and the 22 November 2016, and 35 patients were assigned to each treatment. Prior to the second amendment, 1 patient that had given consent was not included due to an ongoing vasopressor therapy. For a CONSORT flowchart of patients, see Fig. 1. Two patients experienced a suspected allergic reaction following the administration of ^125^I-human serum albumin and were not included in the study. One patient withdrew consent during the intervention. Plasma volume measurement was unsuccessful due to technical problems in 3 patients. A total of 33 patients receiving slow infusion and 31 patients receiving rapid infusion were included in the analysis. Pre-treatment characteristics are presented in Table 1. The two most common criteria for fluid administration were a positive passive leg raising test and an elevated arterial lactate. All patients with a positive passive leg raising test also had at least 1 additional objective criteria suggesting hypovolemia. A total of 4 patients received treatment with norepinephrine at inclusion, and in all cases, the dose was below 0.1 μg/kg/min. Baseline plasma volume was 46.9 ± 9.2 ml/kg and 47.7 ± 6.3 ml/kg in the slow and rapid groups, respectively (Fig. 2). This corresponded to a calculated blood volume of 73.1 ± 15.0 ml/kg and 72.9 ± 10.3 ml/kg in the slow and rapid groups, respectively.

**Table 1:** Demographics

	Slow infusion (*n* = 33)	Rapid infusion (*n* = 31)
Gender (female)	25 (76)	16 (52)
Age, years	69 (65–74)	68 (55–74)
BMI, kg/m^2^	25 (23–30)	25 (23–27)
ASA class 1	0	2 (6)
ASA class 2	22 (65)	21 (68)
ASA class 3	11 (33)	8 (26)
Inclusion criteria		
PLR	23 (70)	20 (65)
ScvO2	10 (29)	9 (29)
Lactate	21 (62)	20 (65)
Urine production	13 (38)	13 (42)
Systolic blood pressure	9 (27)	8 (26)
Operation time, min	368 (289–479)	418 (365–467)
Intraoperative bleeding, ml	600 (300–1000)	500 (300–1000)
Epidural analgesia	29 (88)	30 (97)
Intraoperative fluids		
Crystalloids, ml	4250 (4000–5250)	4250 (3500–5250)
Colloids, ml	500 (500–1000)	700 (250–1000)
Pre-treatment hemodynamics		
HR, beats/min	84 (74–93)	88 (71–97)
Systolic blood pressure, mmHg	114 (96–125)	112 (99–129)
MAP, mmHg	77 (68–90)	77 (68–88)
CVP, cmH_2_O	3 (0–6)	3 (−1–8)
Urine production, ml/kg/h	0.8 (0.4–2.2)	0.8 (0.5–1.0)
Pre-treatment laboratory data		
Albumin, g/l	31 (28–34)	33 (31–35)
Lactate, mmol/l	2.2 (1.7–3.1)	2.7 (1.5–3.2)
ScvO_2_, %	73 (67–76)	73 (68–78)
Hct (%)	36 (34–38)	35 (32–39)
Glycocalyx components and hormones		
Hyaloronan, ng/ml	153 (125–200)	174 (132–208)
Syndekan-1, ng/ml	56 (32–96)	91 (54–190)
Glypican-4, ng/ml	13 (10–17)	13 (10–21)
Copeptin, pmol/l	138 (75–244)	113 (71–207)
MR-proANP, pmol/l	85 (61–106)	94 (71–139)
Renin, mU/l	51 (23–144)	40 (19–131)
Norepinephrine at the time of inclusion	3 (9)	1 (3)

Data are presented as number (percent) or median and interquartile range unless stated otherwise

*Abbreviations*: *BMI* body mass index, *PLR* passive leg raising, *ScvO*_*2*_ central venous saturation, *HR* heart rate, *MAP* mean arterial pressure, *CVP* central venous pressure, *BE* base excess, *ScvO*_*2*_ central venous oxygen saturation, *Hct* hematocrit, *MR-pro-ANP* mid-regional pro-atrial natriuretic peptide

### Outcomes

**Fig. 3:**
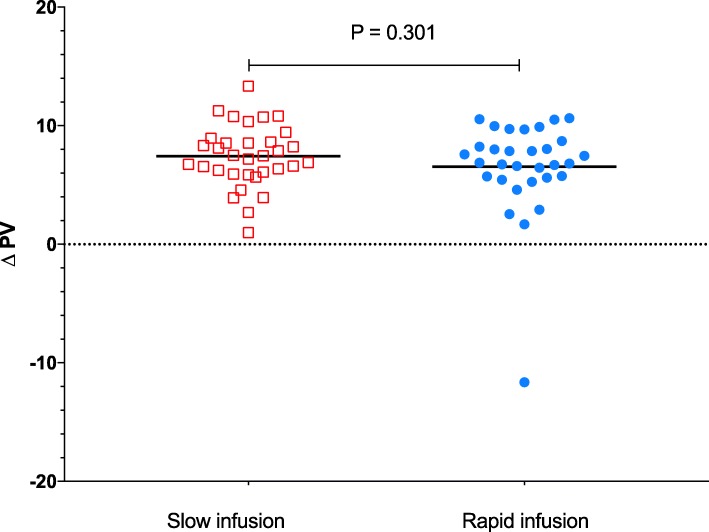
Effect of infusion rate on the change in plasma volume (∆PV) from prior to the start of albumin infusion to 180 min after the start of the infusion of 5% albumin

The increase in plasma volume from the start to 180 min after the start of infusion did not differ between the two infusion rates and was 7.4 ± 2.6 ml/kg and 6.5 ± 4.1 ml/kg in the slow and rapid infusion groups, respectively (mean difference, 0.9 [95%CI, − 0.8 to 2.63.2], *P* = 0.301, *t* test, Fig. 3). Change in the area under the plasma volume curve over time was smaller in the slow infusion group than in the rapid infusion group and was 866 ± 341 min ml/kg and 1226 ± 419 min ml/kg in the slow and rapid groups, respectively (mean difference, 360 min ml/kg [95%CI, 169 to 550], *P* < 0.001, *t* test) (Table 2). The number of patients with postoperative complications in the slow and rapid infusion groups was 8 and 6, respectively, and did not differ between the groups (*P* = 0.774) (Table 2 and Additional file 1: Table S2). No treatment effect on transcapillary escape rate for albumin, lactate, hematocrit, or any of the hemodynamic parameters could be detected (Table 2). Overall diuresis from the start of infusion of albumin to 360 min after the start of infusion did not differ between the treatment groups and was 1.0 ± 0.4 and 1.1 ± 0.6 ml/kg/h (mean difference, − 0.1 ml/kg/h [95%CI, − 0.1 to 0.4, *P* = 0.325, *t* test). In a post hoc analysis, hourly diuresis during this time period was analyzed using a mixed linear regression model and diuresis as measured at 60 to 180 min from the start of the infusion was higher in the rapid infusion group than in the slow infusion group (Additional file 1: Figure S7). A significant interaction between the time period treatment effect was detected suggesting a lower diuresis in the slow group during the earlier hours and higher diuresis at the later periods relative to the rapid infusion group (*P* = 0.002 for interaction between time period and the treatment group, Additional file 1: Figure S7). Plasma albumin concentration increased more in the slow than in the rapid group (Table 2).

**Table 2:** Secondary outcomes

Outcomes	Slow infusion (*n* = 33)	Rapid infusion (*n* = 31)	Absolute difference/risk reduction (95%CI)	*P* value
Hemodynamic outcomes				
Change in plasma volume over time, min ml/kg	866 ± 341	1226 ± 419	360 (169 to 550)	*P* < 0.001
TER, %/h	5.3 ± 3.1	5.4 ± 3.0	0.1 (− 1.6 to 1.5)	*P* = 0.931
ΔHR, beats/min	0 ± 12	1 ± 10	1 (− 7 to 4)	*P* = 0.665
ΔScvO_2_, %	1 ± 5	2 ± 9	1 (− 3 to 5)	*P* = 0.682
ΔHct, %	− 4 ± 2	− 4 ± 2	0 (− 1to 1)	*P* = 0.912
ΔMAP, mmHg	4 ± 12	5 ± 13	1 (− 7 to 6)	*P* = 0.830
ΔCVP, cmH_2_O	2 ± 3	1 ± 3	1 (− 3 to 1)	*P* = 0.164
ΔAlbumin, g/l	4.9 ± 1.6	3.8 ± 1.4	1.1 (0 to 2)	*P* = 0.017
ΔLactate, mmol/l	− 0.4 ± 0.8	− 0.3 ± 1.2	0.1 (− 0.4 to 0.6)	*P* = 0.565
Postoperative complications				
Postoperative complications, number	8	6	2 (−2.2 to 0.5)	*P* = 0.765
Glycocalyx components and hormones				
ΔHyaloronan, ng/mL	− 2.6 (− 29.7 to 19.1)	2.8 (− 26.0 to 51.0)	16.6 (− 52.8 to 19.6)	*P* = 0.461
ΔSyndecan-1, ng/mL	31.1 (− 0.3 to 49.3)	− 1.6 (− 17.0 to 64.4)	2.9 (− 39.1 to 44.9)	*P* = 0.160
ΔGlypican-4, ng/mL	1.2 ± 5.8	− 2.5 ± 7.9	3.6 (0 to 7.3)	*P* = 0.048
ΔCopeptin, pmol/l	− 73.9 (− 140.8 to 26.9)	− 59.3 (− 150.1 to 39.5)	20.6 (− 40.3 to 81.6)	*P* = 0.785
ΔRenin, mU/l	− 14.3 (− 48.0 to − 5.3)	− 29.9 (− 82.8 to − 7.6)	27.4 (− 6.2 to 60.9)	*P* = 0.186
ΔMR-proANP, pmol/l	20.6 (10.5 to 33.2)	47.8 (31.8 to 71.8)	− 25.3 (− 40.0 to − 10.7)	*P* < 0.001

Data are presented as mean ± SD or median and IQR. Fisher’s exact test, unpaired *t*-test, or Mann-Whitney test was used for the analysis as appropriate

*Abbreviations*: *TER* transcapillary escape rate, *Δ* change from baseline to 180 min after the start of infusion, *HR* heart rate, *ScvO*_*2*_ central venous oxygen saturation, *Hct* hematocrit, *MAP* mean arterial pressure, *CVP* central venous pressure, *MR-pro-ANP* mid-regional pro-atrial natriuretic peptide

Plasma concentration of the stable precursor fragment of atrial natriuretic peptide, mid-regional pro-atrial natriuretic peptide, increased more from the start to 180 min after the start of infusion in the rapid infusion group than in the slow infusion group whereas renin and copeptin, the latter reflecting vasopressin release, did not differ between the groups (Table 2). Glypican-4, a component of the endothelial glycocalyx, increased more in the slow infusion group than in the rapid infusion group whereas no difference in the change in hyaluronan and syndecan-1 could be detected (Table 2).

The pre-planned sensitivity analysis did not demonstrate an interaction between baseline blood volume and treatment effect (*P* = 0.075, two-way ANCOVA, Additional file 1: Figure S1) or between the type of surgery and treatment effect (*P* = 0.364, two-way ANCOVA). To further explore if the treatment effect was dependent on baseline blood volume, patients with baseline blood volume above and below the median were analyzed separately, and the results aligned with the ANCOVA results (Additional file 1: Figure S3). The treatment effect in Whipple and gynecological surgery patients was also analyzed separately, and no difference in the primary outcome could be demonstrated in either of the groups (Additional file 1: Figure S5). On a post hoc basis, change in 1-Hct over time was analyzed and was 3.6 ± 1.7 and 6.0 ± 2.6%/min in the slow infusion and rapid infusion groups, respectively (mean difference 2.4, [95%CI, 1.3–3.5%/min, *P* < 0.001, *t* test) (Additional file 1: Figure S4).

## Discussion

In postoperative patients with signs of hypoperfusion, we could not demonstrate that the infusion rate of 5% albumin influenced plasma volume expansion at 180 min after the start of infusion. The change in the area under the plasma volume curve was larger in the rapid infusion group than in the slow infusion group. Infusion rate did not influence vascular leak or hemodynamic parameters. Mid-regional pro-atrial natriuretic peptide concentration increased less in the slow infusion group compared to the rapid infusion group.

The volume of fluid used in the present study is within the range of that used in previous studies investigating the hemodynamic effects of fluid bolus therapy [16, 17, 31–33]. Also, the rate of infusion in the rapid infusion group agrees with that commonly used for a fluid bolus [31–33], whereas the rate of infusion in the slow infusion group was based on previous experimental studies and institutional practice [13, 14]. Fluid boluses are commonly used to correct suspected hypovolemia in hemodynamically unstable patients, and presumed benefits include a rapid correction of hypovolemia. In a recently published survey on global ICU fluid resuscitation practices in 2014, it was reported that about 22% of all fluid resuscitations in surgical ICU patients were performed using albumin [34]. This aligns with a consensus statement on perioperative fluid therapy suggesting the use of both crystalloids and colloids in major surgery [35]. Note that the average patient in the present study had received about 4300 ml of crystalloid and 500 ml of colloid prior to inclusion. Taken together, the above supports the relevance of investigating the physiological response to the administration of albumin.

As mentioned in the introduction, experimental data from a rat sepsis model suggests that plasma volume expansion at 180 min after the start of infusion for a given volume of colloid as well as the area under the plasma volume curve is lower when using a bolus compared to a slower infusion [13, 14]. Our results of no difference in the primary outcome, i.e., change in plasma volume from the start to 180 min after the start of infusion, and a lower area under the plasma volume curve in the slow infusion group indicate that in this clinical setting, a slow infusion, if anything, is inferior to a bolus with regard to plasma volume expansion. The reasons for the differences in the results may include species differences but could also be related to etiology and/or severity of the systemic inflammatory response syndrome reaction.

Plasma volume expansion of 5% albumin as a fraction of infused volume is shown to be in the range of 50–110% immediately after infusion in previous studies using a similar methodology in postoperative or in septic patients [9, 10, 36]. Our results extend these previous findings and show that the volume-expanding effect persists for at least 2.5 h after termination of the infusion. The observation that plasma volume expansion by 5% albumin differs between different studies may in part be explained by the etiology of hypovolemia. Experimental data has shown that albumin is a potent plasma volume expander after a controlled hemorrhage, in which homeostatic mechanisms are intact and may contribute to the restoration of normovolemia [37]. In contrast, albumin is less efficacious as a plasma volume expander in inflammatory conditions in which disruption of homeostatic mechanisms is the cause of hypovolemia [37]. It is tempting to speculate that the variation in plasma volume expansion between individual patients in our dataset could be explained by individual differences in the response to the surgery-induced trauma. It should also be noted that there is a signal for a negative correlation between baseline blood volume and plasma volume expansion in our sensitivity analysis, which could contribute to the variable plasma volume expansion by albumin.

Previous data have shown that transient hypervolemia induced by rapid administration of colloids increases plasma concentration of atrial natriuretic peptide which is suggested to induce shedding of components of the endothelial glycocalyx, which in turn is associated with impairment of endothelial barrier function [18, 38]. We therefore hypothesized that a rapid infusion could induce shedding of glycocalyx components and thereby increase endothelial permeability. However, our results that transcapillary escape rate for albumin was in the normal range and did not differ between the groups, and that there was no increase in plasma concentration of circulating glycocalyx components after rapid infusion, indicate that such an effect is small or non-existent [27, 28]. The difference in the results between our and previous clinical studies with regard to the effects of rapid plasma volume expansion on glycocalyx shedding could be explained by the fact that we aimed to include hypovolemic patients which probably made hypervolemia less likely [18]. Moreover, it is possible that the timing of the measurement of the transcapillary escape rate of albumin may have influenced our results. Interestingly, our post hoc analysis of plasma albumin revealed a larger increase in the slow group at 180 min after the start of the infusion. This result suggests the possibility that extravasation of albumin was lower in the slow infusion group during this time period. If so, this could be explained by the less convective transport of albumin secondary to a lower average intravascular volume from baseline to 180 min in the slow group (lower area under the plasma volume curve). However, the plasma concentration of albumin reflects a balance between synthesis, metabolism, extravasation, and lymphatic return of albumin and therefore is a less reliable marker of vascular leak than radiolabeled albumin.

The tissue trauma induced by major surgery is suggested to initiate a stress response and a systemic inflammatory response syndrome with endothelial dysfunction and disruption of microvascular function as a consequence [39, 40]. The low incidence of vasopressor use in our cohort (Table 1) despite epidural analgesia and the finding that transcapillary escape rate of albumin was in the normal range [26, 27] suggest a preserved endothelial barrier function and a relatively mild systemic inflammatory response. Thus, the generalizability to patients with a more severe disturbance of vascular homeostasis and increased endothelial permeability, such as post-cardiac surgery or in septic shock, is uncertain [27]. Similarly, the results cannot be generalized to other resuscitation fluids such as crystalloids and hyperoncotic albumin solutions or to patients without signs of hypovolemia.

To minimize the radiation dose, the change in the area under the plasma volume curve was estimated by only three plasma volume measurement, and it could be argued that more frequent measurements of plasma volume could have influenced our results. However, the similar result when evaluating changes in plasma volume by analyzing changes in hematocrit every 30 min suggests that a greater resolution in our measurement would not have influenced our conclusions and supports the robustness of our data.

The strengths of the study include the low risk of bias due to the randomized design, publication of the study protocol, the blinded measurement of the majority of the outcomes, and the blinded analysis of the outcomes. Moreover, the use of the gold standard for the measurement of plasma volume [25] and auditing support the reliability and scientific integrity of the data.

## Conclusions

This study does not support our hypothesis that a slow infusion of colloid results in a greater plasma volume expansion than a rapid infusion. Instead, our result of a smaller change in the area under the plasma volume curve indicates that a slow infusion results in a less efficient plasma volume expansion, but further studies are required to confirm this finding. A rapid infusion has no effect on vascular leak as measured after completion of the infusion.

## Additional file

Additional file 1: Supplementary figures and tables. (DOCX 370 kb)

## Abbreviations

ANCOVA: Analysis of covariance
ANOVA: Analysis of variance
ASA: American Society of Anesthesiology
CI: Confidence interval
IQR: Interquartile range
MAP: Mean arterial pressure
PV: Plasma volume
SD: Standard deviation
TER: Transcapillary escape rate
