# Supplementary material for: Albumin infusion rate and plasma volume expansion: a randomized clinical trial in postoperative patients after major surgery
Source: Crit Care. 2019 May 28;23:191. doi: 10.1186/s13054-019-2477-7 (PMC6537197; doi:10.1186/s13054-019-2477-7)
Supplement: Supplementary file 1 — Supplementary figures and tables. (DOCX 370 kb) [file 13054_2019_2477_MOESM1_ESM.docx]

**Additional file 1**

**Plasma volume and blood volume measurements**

Plasma volume was measured at three time points using ^125^I-human serum albumin (HSA) (SERALB-125^®^, CIS Bio International, Gif-Sur-Yvette, France) in a total activity of at most 0.4MBq (corresponding to a dose of 0.12 Gy). The tracer was administered intravenously using syringes containing a known activity of tracer. Blood samples were collected prior to injection of ^125^I-HSA and 10 minutes after injection of ^125^I-HSA. Following collection samples were centrifuged at and plasma was then transferred to pre-weighed vials. Plasma volume was determined by measuring the change in weight of the vial using a precision scale. Plasma activity was then determined in a gamma counter (PerkinElmer 1480 Wizard) by measurement of activity of the plasma samples and plasma volume was calculated by dividing the injected activity of ^125^I-HSA by the change in activity post injection. Injected activities were corrected for remaining activity in the syringes. Plasma volumes were normalized to predicted body weight. For calculation of blood volumes plasma volumes were divided by 1-haematocrit. As suggested by the World Health Organization for low doses of radiation, the thyroid uptake of radioactive iodine was not blocked [1]. The local radiation safety committee approved the protocol.

**References**

**1.** World Health Organization. Protection of the Human Environment (‎1999)‎.

Guidelines for iodine prophylaxis following nuclear accidents: update 1999. Geneva: World Health Organization. <http://www.who.int/iris/handle/10665/66143>

**Table S1**. Definitions of complications

| **Infectious** |  |
| --- | --- |
| Pneumonia | X-ray findings suggesting infiltrates + clinical signs or increase in CRP/temperature + treatment with antibiotics |
| Abdominal infection | Clinical signs + increase in CRP/temperature (>38.5° centigrade) + treatment with antibiotics |
| Wound infection | Clinical signs (rubor, tumor, calor, dolor, functio laesa) + increase in CRP/temperature or positive culture + treatment with antibiotics |
| Urinary tract infection | Leucocytes + nitrate on urine sticks or positive culture + treatment with antibiotics |
| Catheter infection | Local irritation + clinical signs or positive culture + treatment with antibiotics |
| Sepsis | 2 out of 4 SIRS criteria + likely infection + treatment with antibiotics |
| Septic shock | Sepsis necessitating inotropic support |
| Infection with unclear focus | CRP rise + fever + treatment with antibiotics |
| **Cardiovascular** | |
| Myocardial infarction | Increase in Troponin T + one of the following: typical symptoms for at least 15 min or new infarction signs on ECG (Q-wave in at least 2 leads, new LBBB, new ST-T changes) or loss of viable myocardium / new movement anomaly on cardiac ultrasound) |
| Postoperative hypotension | Mean arterial pressure < 65 mmHg despite adequate volume transfusion, necessitating inotropic/vasopressor support |
| New arrythmia | New persistent arrhythmia on ECG necessitating treatment |
| Pulmonary oedema | Clinical signs + x-ray |
| Stroke | New neurological deficit |
| Pulmonary embolism | CT or scintigram |
| Deep vein thrombosis | Ultrasound or angiogram |
| **Respiratory** | |
| Pleural effusion | X-ray or ultrasound |
| Pulmonary embolism | CT scan or lungscintigraphy |
| Prolonged need for respiratory support | Reintubation/NIV |
| Secretions necessitating interventions | Clinical signs + intervention (deep suctioning, extra physiotherapy, NIV, intubation) |
| ALI/ARDS | Sudden onset + bilateral infiltrates on x-ray (in absence of left heart failure) + PaO2/FiO2 < 300/200 |
| **Abdominal** | |
| Prolonged paralytic ileus | No bowel movement > 6 days postoperatively |
| Intraabdominal hypertension | >20 cmH2O surgical intervention necessary |
| Abscess | x-ray + clinical signs |
| Intestinal ischemia | Visual diagnosis during reoperation |
| Anastomostic leakage | Visual diagnosis during reoperation |
| Wound dehiscence | Surgical intervention necessary (in the ward or in theatre) |
| **Renal** | |
| Acute Kidney Injury | According to the KDIGO practice guidelines Kidney Inter. Suppl. 2012 |
| Need for dialysis | Dialysis |
| **Bleeding disorders** | |
| Gastrointestinal bleeding | Clinical signs + pharmacologic or surgical intervention |
| Coagulopathy | PK>1.8 + APTT>60 sec or platelets count < 80.000 |
| Unspecified bleeding | Transfusion of >1 unit of erythrocytes postoperatively |
| **Prolonged stay in PACU or admission to ICU** | Still in PACU after 10.00 a.m. the first postoperative day due to need for prolonged observation |

**Table S2.** Breakdown of complications in respective group

|  | Slow infusion | Rapid infusion |
| --- | --- | --- |
| Patients suffering complications n (%) | 8 (24) | 6 (19) |
| Description of postoperative complications | abdominal infection n = 4  prolonged paralytic ileus n = 3  pleural effusion n = 1 | abdominal infection n = 3  wound dehiscence n = 2  pleural effusion n = 1 |
|  |  |  |

**Figure S1.** Experimental protocol. PV1 (baseline plasma volume), PV2 (plasma volume after 30 minutes), PV3 (plasma volume after 180 minutes, Hct (hematocrit), ScvO_2_ (central venous saturation), BP (blood pressure), CVP (central venous pressure), TD (Diuresis per hour), TER (Transcapillary Escape Rate).

**Figure S2.** Hourly diuresis in the rapid and slow groups from baseline to 360 min after stat of infusion of albumin. Mixed linear regression revealed a significant interaction between time period and treatment effect (P=0.002) and differences in urine output at 120 and 180 minutes. Data are shown as mean ± SD.

**Figure S3.** Plot of change in plasma volume (∆PV) from start to 180 minutes after start of the infusion of albumin in respective treatment groups as a function of blood volume (BV).

**Figure S4.** Change in plasma volume from start to 180 minutes after start of the infusion of albumin in patients with baseline blood volume above or below median. Outcomes within each baseline blood volume were analysed using unpaired t-test without adjustment for multiple comparisons. Horizontal line represents the median.

**Figure S5.** Change in plasma volume from start to 180 minutes after start of the infusion of albumin in which data for patients subjected to Whipple or major gynaecological cancer surgery (Gyn.) are presented separately. Outcomes within each type of surgery were analysed using an unpaired t-test without adjustment for multiple comparisons. Horizontal line represents the median.

**Figure S6.** Plot of 1-Hct from start to 180 minutes after start of the infusion of albumin. Data are presented as mean ± SD. Error bars for the rapid group point up and error bars for slow group point down.

**Figure S7.** Plot of change in plasma activity of ^125^I labelled human serum albumin (^125^I-HSA) relative peak concentration as measured from 190 minutes to 240 minutes after start of the 5% albumin infusion. Data are shown as mean and SD. Error bars for the rapid group point up and error bars for slow group point down.
